# Supplementary material for: Polycystic Ovary Syndrome and the Internet of Things: A Scoping Review
Source: Healthcare (Basel). 2024 Aug 21;12(16):1671. doi: 10.3390/healthcare12161671 (PMC11354210; doi:10.3390/healthcare12161671)
Supplement: Supplementary file 1 [file healthcare-12-01671-s001.zip › Supplementary_File_S2_OVID_MEDLINE_Database_Search_Strategy.pdf]

Database: Ovid MEDLINE(R) ALL <1946 to December 19, 2023>

Search Strategy:

1 Social Media/ (16582)

2 "social media".mp. (37187)

3 facebook.mp. (6908) 4 twitter.mp. (7085)

5 youtube.mp. (4083) 6 instagram.mp. (2077) 7 tiktok.mp. (486)

8 bytedance.mp. (9)

9 snapchat.mp. (236)

10 reddit.mp. (693)

11 substack.mp. (0)

12 linkedin.mp. (389)

13 weibo.mp. (491)

14 wechat.mp. (1243)

15 whatsapp.mp. (1858)

16 or/1-15 (45433)

17 Internet/ (82085)

18 Internet of Things/ (1102)

19 digital.mp. (200212)

20 "digital health".mp. (8810)

21 "digital technology".mp. (3825)

22 (digital adj platform*).mp. (1483)

23 (digital adj technolog*).mp. (6117) 24 "internet of things".mp. (7412)

25 IoT.mp. (6455)

26 "internet of medical things".mp. (386) 27 IoMT.mp. (355)

28 or/17-27 (286305)

29 Social Networking/ (5536)

30 Blogs/ (0)

31 (social adj2 network*).mp. (31851) 32 blog*.mp. (3088)

33 "online post".mp. (121)

34 microblog.mp. (171)

35 (online adj communit*).mp. (1541) 36 (online adj2 platform*).mp. (4817)

37 (content adj creator*).mp. (115)

38 influencer*.mp. (2064)

39 pinterest.mp. (106)

40 clubhouse.mp. (151)

41 threads.mp. (6676)

42 bluesky.mp. (21)

43 mastodom.mp. (0)

44 telegram.mp. (230)

45 or/29-44 (49551)

46 Mobile Applications/ (11994)

47 (mobile adj2 app*).mp. (21453)

48 chat bot*.mp. (39)

49 or/46-48 (21489)

50 Telemedicine/ (38573)

51 tele*.mp. (253667)

52 "mobile phone".mp. (11899)

53 mhealth.mp. (9983)

54 "mobile health".mp. (12995)

55. 55 ehealth.mp. (7454)

56. 56 (remote adj2 consultation*).mp. (6747)

57. 57 zoom.mp. (3853)

58. 58 or/50-57 (281186)

59. 59 Wearable Electronic Devices/ (8266)

60. 60 Monitoring, Ambulatory/ (8664)

61. 61 Monitoring, Physiologic/ (58969)

62. 62 wearable*.mp. (30353)

63. 63 smart electronic*.mp. (216)

64. 64 smartwatch*.mp. (1182)

65. 65 fitness tracker*.mp. (1496)

66. 66 (oura adj2 ring).mp. (49)

67. 67 whoop.mp. (120)

68. 68 fitbit.mp. (1323)

69. 69 garmin.mp. (287)

70. 70 iwatch.mp. (2)

71. 71 apple*.mp. (23381)

72. 72 hexoskin.mp. (25)

73. 73 activity tracker*.mp. (1101)

74. 74 wearable health device*.mp. (43)

75. 75 augmented reality.mp. (4937)

76. 76 health monitor*.mp. (7755)

77. 77 sensor technolog*.mp. (2403)

78. 78 biometric sensor*.mp. (56)

79. 79 smart textile*.mp. (562)

80. 80 or/59-79 (132692)

81. 81 Blood Glucose Self-Monitoring/ (10159)

82. 82 glucose monitor*.mp. (11952)

83. 83 81 or 82 (16528)

84. 84 16 or 28 or 45 or 49 or 58 or 80 or 83 (756317)

85. 85 1 or 17 or 18 or 29 or 30 or 46 or 50 or 59 or 60 or 61 or 81 (225323)

86. 86 exp Polycystic Ovary Syndrome/ (18391)

87. 87 Polycystic Ovar$.ti,ab,tw. (22027)

88. 88 PCOS.ti,ab,tw. (15754)

89. 89 (stein-leventhal or leventhal).tw. (742)

90. 90 (ovar$ adj2 (sclerocystic or polycystic)).tw. (22165)

91. 91 or/86-90 (25492)

92. 92 84 and 91 (198)
