# Supplementary material for: Polycystic Ovary Syndrome and the Internet of Things: A Scoping Review
Source: Healthcare (Basel). 2024 Aug 21;12(16):1671. doi: 10.3390/healthcare12161671 (PMC11354210; doi:10.3390/healthcare12161671)
Supplement: Supplementary file 1 [file healthcare-12-01671-s001.zip › Supplementary_File_S3_Table_of_Results.pdf]

Study ID (Country)	Domain(s)	Aims & Objectives	Study characteristics	Key findings
Alotaibi & Shaman, 2020 [40]<br>and<br>Alotaibi & Alsinan, 2016 [41]<br><br>(Saudi Arabia)	Social media	Evaluate the effectiveness of the PCOS system “Mobile PCOS Management and Awareness System for Gulf Countries” (a private social media network, similar to Twitter/X), described in Alotaibi & Alsinan 2016 [41].<br><br>The PCOS system is aimed at improving the management and awareness of PCOS, and also empower women in gulf countries with relevant knowledge about its management and awareness.	RCT.<br><br>54 women diagnosed with PCOS, aged between 20 and 40 years:<br>- Using PCOS system, n= 22, age (mean ± standard deviation): 26.73 ± 5 years<br><br>- Not using PCOS system, n= 32, age: 29.34 ± 4.84 years	The prototype system was developed in such way to allow its use via web application environment, iPhone, and Android, and was able to carry out all the functional requirements.<br><br>The awareness levels of those using the PCOS system increased to a greater extent compared to that of those not using the PCOS system.<br><br>Medical staff conveyed more than 80 posts and 10 pictures containing data about PCOS.<br><br>Approximately 120 text messages between medical staff and the patients were refined.<br><br>High dropout rate (nearly 40%).
Ajil *et al.*, 2023 [63]<br><br>(India)	Machine learning; Mobile apps	Explore the feasibility of developing a model that utilizes machine learning algorithms and techniques capable of	Development of a mobile app.<br><br>Dataset from Kaggle, comprised of 543 people and 42 features, including:	The algorithm XG Boost obtained the highest accuracy.

Study ID (Country)	Domain(s)	Aims & Objectives	Study characteristics	Key findings
		automating the diagnosis of PCOS.<br><br>Evaluate the performance of various algorithms, including:<br>- AdaBoost,<br>- DT,<br>- RF,<br>- XG Boost,<br>- hybrid algorithm.<br><br>Uncover meaningful patterns and relationships within the data.	- metabolic,<br>- imaging,<br>- hormonal,<br>- biochemical parameters.<br><br>PCOS: 179 people.<br><br>No PCOS: 364 people	A mobile app was built using the Flask framework, developed in Android Studio, and employed Retrofit for API calls.<br><br>The app assists users in early-stage PCOS prediction, advising them to visit a doctor to seek appropriate treatment.
Atigan & Atigan, 2023 [68]<br><br>(Turkey)	Social media	Analyse the content of PCOS-related exercise videos on YouTube to reveal the quality of the video content.<br><br>Video scoring system:<br>- DISCERN score,<br>- Global quality score,<br>- Video power index.	Content analysis.<br><br>Social Media: YouTube<br><br>YouTube videos, n= 198<br><br>Search term used: ‘PCOS exercise’.	Videos originating from India (n=91) were almost equal to the sum of USA-Canada and Europe-UK videos (n=90): India, n= 91 (46%), European countries and UK, n= 36 (18.2%), USA and Canada, n= 54 (27.3%), other countries, n= 17 (8.6%).<br><br>Exercise type: Yoga, n= 58 (29.3%), Strength training, n= 44 (22.2%), Aerobic exercise, n= 38

Study ID (Country)	Domain(s)	Aims & Objectives	Study characteristics	Key findings
				(19.2%), Combination of at least two of those exercises, n= 58 (29.3%).<br><br>Profiles of the video uploaders: Health employee, n= 28 (14.1%), Nutritionist, n= 25 (12.6%), Sports trainer, n= 48 (24.2%), Patient, n= 21 (10.6%), Undefined, n= 76 (38.4%).<br><br>Most of the videos (n= 133, 67.2%) were uploaded after the COVID-19 pandemic.<br><br>Parameters highlighted along with exercise: Hormonal levels, n= 85 (42.9%); diet, n= 74 (37.4%); Insulin resistance, n= 71 (35.9%); menstruation, n= 52 (26.3%); fertility, n= 31 (15.7%); citing a scientific article, n= 17 (8.6%).
Authier *et al.*, 2020 [90]<br><br>(France)	Websites	Explore the self-reported experiences and disease perceptions posted on Internet discussions forums by women with PCOS and fertility issues.	Qualitative evaluation of online forums.<br><br>Search engine used: Google<br><br>Language: French	785 comments from 211 women on seven open-access French-speaking internet forums.<br><br>Discussion forums appear to be beneficial, providing a source of support and experiential knowledge sharing between those with PCOS.

Study ID (Country)	Domain(s)	Aims & Objectives	Study characteristics	Key findings
				Infertile women with PCOS who consult internet forums appear to have low understanding of the disease, which affects their quality of life.<br><br>Medical information provided to them is perceived as inadequate or insufficient, while anxiety and psychological distress are frequently omitted by health practitioners.
Barrera *et al.*, 2023 [87]<br><br>(USA)	Machine learning	Identify and summarize the body of evidence related to the utility of artificial intelligence and machine learning in diagnosing PCOS.	Systematic review.<br><br>Databases were searched from the time of inception to May 2019.<br><br>Modified version of the QUADAS-2 tool was used.	31 studies met the inclusion criteria and almost half (48%) of them used ultrasound images. Studies were mainly conducted in India (29%) and China (16%) and the sample size ranged from 9 to 2,000 people with PCOS.<br><br>The most common machine learning techniques employed were:<br>- support vector machine (42%, n= 13),<br>- K-nearest neighbor (26%, n= 8),<br>- regression models (23%, n= 7).<br><br>Only a third of studies (32%, n= 10) used standardized PCOS diagnostic criteria (NIH, Rotterdam, or Revised International PCOS classification) as a reference to evaluate the results.

Study ID (Country)	Domain(s)	Aims & Objectives	Study characteristics	Key findings
Bouchard *et al.*, 2023 [52]<br><br>(Canada)	Mobile apps	Establish external validity for the Mira monitor for use in women with regular menstrual cycles.<br><br>Compare those with regular cycles to oligomenorrheic women with PCOS and oligomenorrheic athletes.	Protocol for a prospective cohort study with a longitudinal follow-up.<br><br>Participants will record menstrual cycle observations and Mira Fertility Monitor observations on the *Read Your Body* app.	N/A
Boyle *et al.*, 2018 [58]<br><br>(Australia)	Mobile apps	Determine if women with PCOS identified a need for PCOS-related mobile health apps.<br><br>Also, to evaluate currently available apps.	Community-based study using a combination of:<br>- National survey of women with PCOS (n= 264) to investigate their electronic/mobile health needs.<br><br>- Review of mobile apps (n= 16) for women with PCOS available on the iOS and Android platforms.	Almost all respondents (98%) had a smartphone, and 72% previously used an app to manage their health (46% was PCOS-related). 86% reported that they would be more likely to use an app than a website to help manage PCOS, and 91% would use a PCOS-specific app, if available.<br><br>Most important features in a PCOS-specific app are evidence-based information (95%), ability to record symptoms (95%), opportunities to ask questions to an expert (86%), set health-related goals (83%), having a question prompt list, i.e. a structured list of questions to facilitate communication between them and HCPs (83%).

Study ID (Country)	Domain(s)	Aims & Objectives	Study characteristics	Key findings
				Eight included apps were free, and the other eight cost between AUD$1.29 and AUD$7.99
Chauhan *et al.*, 2021 [66]<br><br>(India)	Machine learning; Mobile apps	Development of an app for the early prediction of PCOS using machine learning techniques.	Comparative analysis.<br><br>Data collected through survey:<br><br>Participants (n= 267):<br>- PCOS, n= 61<br>- Non-PCOS, n= 206<br><br>Age: from <18 to >45, majority (56.7%) between 18-25 years<br><br>27 features collected, including age, weight, cycle length, period length, body hair growth, physical fitness, eating habits.	The algorithms tested comprised of five models:<br>- Naïve Bayes Classifier,<br>- K-Nearest Neighbors,<br>- Support Vector Machine,<br>- Decision Tree Classifier,<br>- Logistic Regression.<br><br>The app was developed based on Decision Tree Classifier, the best performance machine learning model, with 81% accuracy, 70% precision, and 94% specificity.<br><br>Through the user's answers, the app predicts PCOS diagnosis and suggests the user to consult a clinician to get adequate treatment.
Chiu *et al.*, 2018 [46]<br><br>(Australia)	Websites	Evaluate the target audience, characteristics, quality, information content, user-friendliness,	Systematic search and assessment of websites with PCOS content.	Majority of websites were commercial (41%, n= 31) and targeted women only (81%, n= 61). These two categories had the lowest quality scores.

Study ID (Country)	Domain(s)	Aims & Objectives	Study characteristics	Key findings
(plus Htet *et al.*, 2018) [47]		and readability of online information on PCOS.<br><br>Content sub-analysis by Htet *et al.*, 2018:<br><br>Assess the accuracy of the information on PCOS-related websites regarding aspects of lifestyle management: diet, physical activity, and weight management.	Separate searches (in Australia and the UK), for webpages describing PCOS information in English, using Google, Bing, and Yahoo.<br><br>The first two pages of search results were compiled, and 75 websites were evaluated and categorised.<br><br>15 websites were eligible for the content sub-analysis by Htet *et al.*, 2018.	Other webpages from: non-profit organizations (32%, n= 24), professional society (15%, n= 11), government (12% n= 9).<br><br>Highest quality scores from professional society websites, and websites target at healthcare providers.<br><br>Content sub-analysis by Htet *et al.*, 2018:<br>Webpages from: commercial (n= 5), professional society (n= 5), non-profit (n= 3), and government (n= 2).<br><br>Lifestyle information on these 15 PCOS websites was limited, and the accuracy of this information was suboptimal.<br><br>These websites lacked specificity and applicability.<br><br>Women with PCOS may be accessing insufficient and likely ineffective health information on websites. This paucity could therefore limit their success at implementing optimal PCOS lifestyle recommendations into their day-to-day and real-world circumstances.

Study ID (Country)	Domain(s)	Aims & Objectives	Study characteristics	Key findings
Choi *et al.*, 2023 [43]<br><br>(plus Lee & Lee, 2023) [42]<br><br>(Korea)	Mobile apps	Choi *et al.*, 2023 [43]:<br>To develop an engaging mobile app based on needs assessment and analysing current evidence for lifestyle modifications in women with PCOS.<br><br>Lee & Lee, 2023 [42]:<br>Assess the effectiveness of a 12-week integrated mobile app (Choi et al. 2023) [43] for lifestyle modifications designed for those who are overweight and have PCOS.	Development study by Choi *et al.*, 2023 [43] - systematic review and survey, followed by Android app design and test.<br><br>Survey participants (n= 30): women with PCOS attending a university hospital in Incheon, Korea.<br><br>Age: 25.17 ± 4.24 years,<br>BMI: 24.2 kg/m^2 (mean).<br><br>Testing the app by Lee & Lee, 2023 [42]:<br>Intervention group (n= 14) – 12-week lifestyle modification program delivered through a mobile app:<br>Age: 28.50 ± 4.83 years<br>BMI: 28.53 ± 4.0 kg/m^2<br><br>Control group (n= 14) – received leaflet with evidence-based information on PCOS	Choi *et al.*, 2023 [43]:<br>None of the respondents had heard of or used a mobile app for lifestyle modification before.<br><br>An Android app was designed based on the contents identified in the analysis phase. The app menus consisted of a diet and exercise diary, daily body weight, questionnaires regarding hirsutism and acne, menstrual period, educational resources with disease and management information, communication with researchers, goal achievement score.<br><br>Lee & Lee, 2023 [42]:<br>After 12 weeks there was significant weight loss in the intervention group compared to the control group (3.19 vs. 0.79 kg, respectively; $p < 0.05$)<br><br>Significant improvements were also observed across time in postprandial insulin levels (22.25 vs. 9.29 μIU/mL, respectively), hirsutism (2.98 vs. −0.16 points, respectively), and depression (7.73 vs. 1.7 points, respectively) between the two groups (all p-values < 0.05).

Study ID (Country)	Domain(s)	Aims & Objectives	Study characteristics	Key findings
			and were instructed to maintain usual lifestyle.<br>Age: 25.36 ± 4.40 years<br>BMI: 27.79 ± 3.05 kg/m^2	Mobile applications for lifestyle modifications offer a promising avenue for addressing the unique challenges faced by women with PCOS and provide accessible and personalized support for their health needs, but further research is needed to explore long-term effects and incorporation of advanced technologies to enhance PCOS management.
Clarke *et al.*, 2023 [69]<br><br>(Jamaica)	Social media	To identify knowledge gaps, improve patient education, and keep a record of high-quality content to facilitate patient education, the study assessed the quality and reliability of content related to PCOS on YouTube by analysing the:<br>- DISCERN score,<br>- Global quality score,<br>- Video power index.	Cross-sectional observational study.<br><br>YouTube videos, n= 80<br><br>Keywords used: PCOS, polycystic ovarian disease, PCOS treatment, PCOS prevention, PCOS cause, and PCOS diet.	Total number of views: n= 36,437,534. Total number of likes: n= 794,779. Total number of comments: n= 54,162.<br><br>Most videos were uploaded by non-physicians (37%, n= 30). 28.8% of the videos were posted by doctors (n= 23), 16.3% by hospitals (n= 13), and 5% by healthcare organizations (n= 4).<br><br>Compared to videos posted by hospitals, videos uploaded by patients (8.8%, n= 7) had a higher popularity (video power index) and lower global quality score.

Study ID (Country)	Domain(s)	Aims & Objectives	Study characteristics	Key findings
Dietz de Loos *et al.*, 2021 [48]<br><br>(plus Jiskoot *et al.*, 2020a, 2020b, and 2017) [49-51]<br><br>(The Netherlands)	Phone-based	Examine whether a three-component intervention was effective to decrease weight compared with usual care in women with PCOS.<br><br>Evaluate whether including text message in the intervention was effective in supporting behavioural change and sustainable weight loss.	RCT.<br><br>Feedback through text message.	The three-component lifestyle intervention showed statistically significant and clinically relevant improvements in metabolic health.<br><br>Compared to the control group, the decrease in the continuous metabolic syndrome severity z-score was greater in the group with text message (after 1 year).<br><br>High dropout rate.
Dmitrovic *et al.*, 2011 [84]<br><br>(Croatia)	Wearables	Assess whether ambient hyperglycemia early in their pregnancy that progressively exacerbates with advancing pregnancy is present in pregnant women with PCOS (measured by continuous glucose monitoring).	Case-control study (singleton pregnancies).<br><br>Continuous glucose monitor (Sensor inserted into the abdominal interstitial tissue and a pager-sized monitor that is worn on the belt).<br><br>PCOS group: n= 17<br>Age: 29 ± 4 years<br>BMI: 32 ± 8 kg/m^2	Serial 24-hour glucose monitoring did not reveal changes in glucose metabolism.<br><br>Hyperandrogenemia improved with progressive pregnancy in women with PCOS.<br><br>Compared with the control group, women with PCOS had a higher area under the curve for glucose during the OGTT at visit 1, 2, and 3, and for insulin at visit 1.

Study ID (Country)	Domain(s)	Aims & Objectives	Study characteristics	Key findings
			Control group: n= 17<br>Age: 31 ± 5 years<br>BMI: 26 ± 7 kg/m^2	Eight women with PCOS (47%) and two women in the control group (12%) developed gestational diabetes mellitus.<br><br>Difference in birth weight between groups was not significant.
Elhariry *et al.*, 2022 [70]<br><br>(UK)	Social media	Establish the demographics and experiences of the top 100 PCOS influencers and organizations advocating for PCOS on Twitter/X.	Semi-structured interviews.<br><br>List of the top 100 PCOS influencers and organizations extracted from Twitter/X using network analysis tools (Cronycle, Symplur, and SocioViz).<br><br>Eight influencers completed a 15-minute semi-structured interview (UK, n= 5; and USA, n= 3).	Of the top 100 influencers, 95% were from high income countries (mainly USA, n= 49; UK, n= 22; Canada, n= 9) and 73.2% (n= 71) were female, while 26.8% (n= 20) were male (plus gender unknown, n= 3).<br><br>The eight influencers who completed the semi-structured interview reported that the motivation and most common reasons why they decided to become involved with PCOS awareness were:<br>- Spread of misinformation (n=7),<br>- Lack of support and correct information available for women diagnosed with PCOS (n=6),<br>- Misconceptions of PCOS impacts on health (n=5),<br>- Misconceptions on the ability to make changes to better one's lifestyle (n=5)

Study ID (Country)	Domain(s)	Aims & Objectives	Study characteristics	Key findings
				Platforms used (some use more than one) were Twitter/X (n=5), own website (n= 4), blog (n= 3), Instagram (n= 3), Facebook (n=2), TikTok (n= 1), WhatsApp group (n= 1), and Clubhouse (n= 1).<br><br>They posted content related to wellbeing (n=6), lifestyle advice (n= 4), recommended diets and nutrition (n= 3), and their experiences in different aspects in relation to PCOS (n= 3).<br><br>The majority of the top 100 organizations (n= 80) worked in high income countries, predominantly from the USA (n= 38) and the UK (n= 27). Those organizations were mainly charities and networks (n= 25), businesses (n= 34), healthcare practices / professionals (n= 28).
Emanuel *et al.*, 2023 [76]<br><br>(New Zealand)	Machine learning; Social media	Explore the feasibility of gathering and analysing laboratory test results posted online by users of the social media platform Reddit (specifically the PCOS subreddit) using machine learning.	Dataset: posts and comments with self-reported laboratory test results, gathered from the PCOS subreddit hosted on the social media platform Reddit, using Pushshift Reddit Dataset (an archive of all platform content since 2015, intended for research use).	Results from the subreddit dataset were broadly consistent with a PCOS population, implying that this self-reported data can be used for further research.

Study ID (Country)	Domain(s)	Aims & Objectives	Study characteristics	Key findings
			PCOS subreddit dataset:<br>Age: 25.5 ± 5.3 years (n= 661)<br>BMI: 24.4 ± 6.4 kg/m^2 (n= 240)<br><br>Comparison: Ten papers were chosen to represent published PCOS literature, with the selection criteria including:<br>- Rotterdam diagnostic criteria for PCOS,<br>- Published within the last 20 years,<br>- Including at least 50 participants with PCOS.<br><br>Literature dataset (n= 2843):<br>Age: 26 ± 26.6 years<br>BMI: 27.4 ± 28.3 kg/m^2	
Hohmann-Marriott *et al.*, 2023 [54]	Mobile apps	Understand perspectives of providers and patients in Aotearoa, New Zealand on the potential benefits, concerns, and	Online qualitative survey and online focus groups.<br><br>Survey: n= 144<br>Age range: 19-55 years.	94% (n= 136) had knowledge and/or experience with one or more of menstruation, endometriosis, PCOS, infertility, and peri/ menopause.<br>49% (n= 71) used a menstrual app before for keeping a record of their menstrual cycle or to

Study ID (Country)	Domain(s)	Aims & Objectives	Study characteristics	Key findings
(New Zealand)		role of menstrual tracking apps and menstrual disorders apps in healthcare.	92% identified their gender as female.	assist in the management of a menstrual disorder. Respondents felt app calendars and tracking improves communication with HCPs.<br><br>They expressed concerns about inaccuracies, how their data may be used, and felt that apps specific to their region, menstrual disorders, diseases, and life stages is needed.
Jain *et al.*, 2021 [56]<br><br>(USA)	Mobile apps	Use the *Flo* app to:<br>- understand characteristics of PCOS across several countries (USA, UK, India, Philippines, and Australia).<br>- identify contributing risk factors.	Analysis of data related to PCOS symptoms, collected from users of the *Flo* mobile app.<br><br>USA: n= 243,238.<br>- Age: 27.3 ± 5.97 years.<br><br>UK: n= 68,325.<br>- Age: 29.7 ± 5.97 years.<br><br>India: n= 40,092.<br>- Age: 25.02 ± 4.71 years.<br><br>Philippines: n= 35,131.<br>- Age: 24.7 ± 5.45 years.	The most prevalent predictors of PCOS were bloating, high cholesterol and/or high glucose.<br>As BMI increased, the percentage of women who reported a physician confirmed PCOS diagnosis also increased. However, women in India did not follow this trend.<br><br>Women with severe obesity in the UK show over three times increase in odds of having a PCOS diagnosis (compared to those with normal weight) (OR: 3.2; $p < 0.05$).<br><br>Bloating was the most commonly reported symptom by women in the USA (73.8%), UK (78.6%), Australia (80.4%), and Philippines (75.4%).

Study ID (Country)	Domain(s)	Aims & Objectives	Study characteristics	Key findings
			Australia: n= 29,926,<br>- Age: 27.28 ± 5.77 years.	
Jeswani *et al.*, 2023 [64]<br><br>(India)	Machine learning; Mobile apps	Create a femtech platform (mobile app: MonAmie) to raise general awareness about menstruation.<br><br>Discuss the findings from the platform development and the results from the comparisons among machine learning algorithms used in its development.	Machine learning for the development of a mobile app (MonAmie).<br><br>PCOS dataset (n= 541) collected from 10 hospitals located near Kerala, India.<br><br>Dataset details: NR<br><br>Classifiers implemented on the dataset:<br>- Decision tree,<br>- SVM,<br>- Random Forest,<br>- Logistic Regression,<br>- KNearest Neighbours,<br>- XGBRF,<br>- Catboost Classifier.	Catboost Classifier achieved the highest accuracy (98.41).<br><br>The PCOS suggester takes in the input from the user and predicts whether the person has PCOS or not using Logistic Regression.<br><br>Components of the app MonAmie:<br>- Period tracker,<br>- Doctor Appointment Schedules,<br>- Awareness,<br>- Fitness and Exercises,<br>- Products (E-Commerce),<br>- PCOS: Tracker and Suggestor.
Karia *et al.*, 2023 [65]	Machine learning;	Provide a platform with accurate PCOS information and can assist	Machine learning for the development of a mobile app (BeRedy).	Accuracy of machine learning algorithms to predict PCOS diagnosis:<br>- Random forest (90.44%),

Study ID (Country)	Domain(s)	Aims & Objectives	Study characteristics	Key findings
(India)	Mobile apps	in the early detection of PCOS.	Seven papers were described in a literature review of studies using image processing and machine learning for diagnosing PCOS.<br><br>The machine learning model for the apps' PCOS diagnosis prediction was tested using:<br>- Linear regression,<br>- K-nearest neighbors,<br>- Random forest.	- Decision tree (80.15%),<br>- K neighbors (72.06%).<br><br>Random forest classifier was used for the PCOS diagnosis predictor of the app, which also includes a menstrual cycle tracker, a chatbot, and menstrual awareness blogs.
Liu *et al.*, 2022 [60]<br><br>(China)	Mobile apps; Social media	Investigate the clinical effectiveness of an online and offline health management model in patients with PCOS.	RCT.<br><br>Treatment group (n= 41) received online and offline health management model for 6 months (using Mint Health app and WeChat):<br>Age: 30.5 ± 3.1 years<br>BMI: 27.7 ± 4.9 kg/m^2	The intervention group had statistically lower (P<.001) scores for TCM syndrome, acne, and LH / FSH ratio. After 3-months, the TCM syndrome curative effect index was markedly higher (97.30% vs 54.05%; P<.001) in the intervention group.<br><br>Family intervention supported the patients' self-management ability to improve:<br>- diet management (including a substitute tea drink),<br>- exercise management (including acupoint massage),

Study ID (Country)	Domain(s)	Aims & Objectives	Study characteristics	Key findings
			Control group (n= 41) completed outpatient health education:<br>Age: 29.9 ± 3.5 years<br>BMI: 26.6 ± 4.2 kg/m^2	- standardised medications.
Malhotra *et al.*, 2023 [96]<br><br>(India)	Phone-based	Evaluate the efficacy of an individualised lifestyle intervention compared to usual care in Indian women with PCOS who wish to conceive.	Protocol for a multicentre RCT.<br><br>Once a week text messages and/or videos on diet and physical exercise.<br><br>Once a month telephonic contact to assess diet/exercise compliance.	N/A
Malhotra *et al.*, 2023 [71]<br><br>(UK)	Social media	Study the digital impact of PCOS Awareness Month initiative.<br><br>Identify the common themes and associated topics during the awareness month.	Serial cross-sectional analysis.<br><br>Total number of tweets and impressions for PCOS per day, from August 1st to October 31st 2014-2022 (September is PCOS Awareness Month).	In September 2020, the highest spike of total tweets (16,465) for PCOS hashtags was noted, representing the highest yearly increase (136.1%) in total tweets since September 2014. This coincided with the increased global online activity due to the pandemic.

Study ID (Country)	Domain(s)	Aims & Objectives	Study characteristics	Key findings
		Identify the top influencers during the awareness month.<br><br>Study the global equity of influence during the awareness month.	The hashtags #PCOS, #PCOSawarenessmonth, and #PCOSawareness were tracked.	PCOS received 120,828 million and 199,13 million impressions in September 2021 and 2022, respectively.<br><br>The top 10 most influential accounts in September 2021 were PCOS researchers and/or advocates (n= 5) and organizations (n= 5). In 2022, the top 10 most influential accounts here PCOS researchers and/or advocates (n= 4) and organizations (n= 6), with seven of the top 10 users being the same in both years.<br><br>The countries with most engagement were USA, UK, Australia, India, Canada, South Africa, and Trinidad & Tobago.<br><br>There was limited engagement in African, Asian, South American, and non-English speaking European countries.
Malhotra & Kempegowda 2023 [72]<br><br>(UK)	Social media	Study the context of YouTube comments about PCOS, find associated themes and identify trends over time.	Big data "infodemiology" study.<br><br>85,872 comments fetched and analysed from 940 videos published on YouTube with	From 13,106 comments where a specific gender was identified, n= 11,601 (88.5%) were from female users, whereas n= 1506 (11.5%) were by male users.

Study ID (Country)	Domain(s)	Aims & Objectives	Study characteristics	Key findings
		Identify the underlying sentiments and gender-based differences in these YouTube comments.	"PCOS" OR "polycystic ovary syndrome" in the title or description from May 2011 to April 2023.	Key themes associated with female users included symptoms of PCOS (irregular periods and acne), positive personal experiences (such as helpful and love), negative personal experiences (fatigue and pain), motherhood (such as infertility and trying to conceive), self-diagnosis, and the use of professional terminology detailing their journey.
Mallappa Saroja & Chandrashekar 2010 [95]<br><br>(UK)	Websites	Evaluate the quality of patient information on PCOS found on websites through search results using the most commonly used search engines.	Content analysis.<br><br>Search engines used: Google, Yahoo, MSN, Ask, AOL (only results in English were included).<br><br>Keywords used: PCOS, patient information.	15 websites included, originating from: USA (n= 11), UK (n= 3), and Australia (n= 1).<br><br>All websites gave adequate explanation about signs and symptoms, but only two correctly described the criteria for diagnosis.<br><br>Thirteen websites mentioned adequate investigations.
Mario *et al.*, 2017 [78]<br><br>(Brazil)	Wearables	Examine the effect of habitual physical activity on anthropometric profiles, BMI, metabolic markers, and hormonal variables of women with PCOS.	Cross-sectional study.<br><br>Digital pedometer (Waistband, BP 148 Techline, Sao Paulo, Brazil).<br><br>PCOS group: n = 84<br>Age: 24.6 ± 5.8 years	Active women with PCOS had a better anthropometric and metabolic profile than sedentary women with PCOS of the same age.<br><br>An increment of 2000 steps per day in habitual physical activity was independently associated with decreased free androgen index.

Study ID (Country)	Domain(s)	Aims & Objectives	Study characteristics	Key findings
			BMI: 29.4 ± 5.9 kg/m^2<br><br>Control group: n = 67<br>Age: 26.4 ± 5.4 years<br>BMI: 28.4 ± 5.9 kg/m^2	
Mario *et al.*, 2012 [82]<br><br>(Brazil)	Wearables	Assess total and segmental lean muscle mass in women with classic and ovulatory PCOS (compared to controls).<br><br>Verify if lean mass is associated with hormone and metabolic features in women with classic and ovulatory PCOS.	Age-matched case-control.<br><br>Digital pedometer (Waistband, BP 148 Techline, Sao Paulo, Brazil).<br><br>Classic PCOS group: n= 30<br>Age: 23.4 ± 5.8 years<br>BMI: 32.5 ± 5.8 kg/m^2<br><br>Ovulatory PCOS group: n= 13<br>Age: 22.9 ± 7.3 years<br>BMI: 27.0 ± 3.6 kg/m^2<br><br>Control group: n= 22<br>Age: 27.2 ± 6.3 years<br>BMI: 27.6 ± 6.6 kg/m^2	BMI, waist circumference, systolic blood pressure, and metabolic profile were higher in classic PCOS.<br><br>Increase in trunk and total lean mass in classic PCOS appears to be associated with insulin resistance and central obesity – rather than with energy intake, physical activity or androgens.
Michael *et al.*, 2015 [79]	Wearables	Examine physical activity patterns and predictors of	Cross-sectional study.	Most (60%) participated in at least a 10-minute bout of MVPA each day. Only 14% averaged bouts

Study ID (Country)	Domain(s)	Aims & Objectives	Study characteristics	Key findings
(USA)		moderate to vigorous physical activity in younger people with PCOS, using a multisensor activity monitor.<br><br>Examine BMI and depressive symptoms as predictors of physical activity in younger people with PCOS.	Activity tracker (SenseWear Pro armband, Body Media Inc, Pittsburgh, PA, USA).<br><br>Sample size: n= 35<br>Age: 15.4 years (min: 12; max: 21 years)<br>BMI: 38.1 kg/m^2 (min: 21.7; max: 69.3 kg/m^2)	of 30 or more minutes and BMI was negatively correlated with bout duration (p= 0.04).<br><br>Overall, participants showed a preference for bouts of activity lasting at least 5 to 10 minutes, but less than 30 minutes.<br><br>The total average minutes of daily MVPA were greater than the minimum recommended in the USA guidelines for children.
Mousiolis *et al.*, 2012 [94]<br><br>(Greece)	Websites	Explore what people with PCOS can access on the internet, and hence what background knowledge, misconceptions, and expectations they may have.<br><br>Evaluate the most commonly visited websites for quality of their PCOS content.	Content analysis.<br><br>Search engines used: Google, Yahoo, Bing, Ask (only results in English were included).<br><br>Keywords used (PCOS, polycystic ovaries syndrome, PCOS symptoms) were selected by asking 10 pilot patients.	Data from 15 websites analysed, using a predefined 10-item checklist to evaluate the content of each website included symptoms, diagnosis, treatment, etc. Top ranked websites: Mayo Clinic (10/10), Patient.co.uk (9/10), WebMD (9/10), WomensHealth.gov (9/10).<br><br>Most websites had a Facebook page (n= 13) or a Twitter account (n= 9), but only a few (Facebook: n= 4, Twitter: n= 2) had a social media account specifically dedicated to PCOS.

Study ID (Country)	Domain(s)	Aims & Objectives	Study characteristics	Key findings
		Social media expansion of those websites was also assessed.	Popularity was checked by using Alexa's Traffic Ranks.	
Naroji *et al.*, 2023 [73]<br><br>(USA)	Social media	Assess the content, engagement, and extent of PCOS-related information on TikTok, Instagram, and Reddit.	Cross-sectional analysis.<br><br>100 posts collected from TikTok and Instagram each, by searching for the term "PCOS" and viewing the top results.<br><br>22,641 posts from the subreddit r/PCOS on Reddit, from the two-year period (June 22, 2020, to June 22, 2022).	Average of 1.8 million views for PCOS related content on TikTok.<br><br>Weight was mentioned the most on TikTok, and diet was mentioned the most on Instagram.<br><br>"Ovarian cysts" and "oral contraceptive pill" - were discussed the least on TikTok and Instagram, respectively.<br><br>Approximately 30% of posts mentioned interactions with healthcare providers.<br><br>Conflict of interest (such as purchasing supplements or signing up for health coaching advertised by the influencer posting) was present in 45% of TikTok posts and 89% of Instagram posts.<br><br>On Reddit (n= 22,641), the average post was 142 words long and received 8.83 comments.

Study ID (Country)	Domain(s)	Aims & Objectives	Study characteristics	Key findings
				"Symptom management" received the most comments, followed by "community experiences".<br><br>Posts about "weight management", "healthcare providers", and "general questions" received the fewest comments.
Peven *et al.*, 2023 [55]<br><br>(UK)	Mobile apps	Evaluate agreement between clinicians and three symptom checkers for: endometriosis, uterine fibroids, and PCOS.	Clinical vignettes study using *Flo.*<br><br>UK-based GPs (n= 5) were recruited to independently create clinical 24 case vignettes of simulated users per condition.	Exact matches between the vignette classification and the symptom checker outcome for PCOS: 88% (n=21).<br><br>For each PCOS symptom checker, sensitivity was reported as 100% and specificity was reported as 75%.<br><br>Positive predictive value for PCOS was reported as 80% and negative predictive value was reported as 100%.
Rodriguez *et al.*, 2020 [67]<br><br>(USA)	Machine learning; Mobile app	Develop and pilot test a predictive model that generated a PCOS risk score: the irregular cycle feature in the menstrual tracking app Clue.	Literature review and virtual tool functionality assessment.<br><br>Data collected through the app Clue (BioWink GmbH).	Compared with the physician score:<br>- First iteration produced one false positive and had an absolute mean difference of 15.5% (SD 15.1%) among the virtual test subjects.

Study ID (Country)	Domain(s)	Aims & Objectives	Study characteristics	Key findings
		Run that model (using 9 virtual test subjects) and:<br>- create a quantitative risk score,<br>- compare it with that of a clinician (board-certified reproductive endocrinology and infertility physician-scientist),<br>- determine the sensitivity and specificity of the model.	Clue users are prompted to input age, height, and weight in order to create their profile, and the app collects information including:<br>- menstrual cycle<br>- length,<br>- duration of flow,<br>- menstruation-related pain symptoms,<br>- method of birth control.	- Second iteration produced 2 false positives and had an absolute mean difference of 18.8% (SD 13.6%).<br><br>A significant positive correlation existed between the feature and physician score (Pearson correlation coefficient=0.82; P=.01).<br><br>The second iteration performed worse, with a Pearson correlation coefficient of 0.73 (P=.03).<br><br>Despite higher probability than the physician's prediction, the irregular cycle feature still proved useful by advising the individual to seek a healthcare provider for further testing.
Sanchez & Jones, 2016 [93]<br><br>(USA)	Websites	Explore PCOS-related content in digital teen and women's magazines and understand how they portray people with PCOS.	Content analysis of teen and women's digital magazines.<br><br>Data from the Alliance for Audited Media (USA) was used to identify popular (circulation rates ≥1,000,001) digital teen and women's magazines.	Three main themes identified, related to:<br>- women's social roles and responsibilities,<br>- personal responsibility to improve health,<br>- use of personal experience with PCOS to advocate for women's health.<br><br>PCOS was depicted as a barrier to childbearing, starting a family, and effective breastfeeding.

Study ID (Country)	Domain(s)	Aims & Objectives	Study characteristics	Key findings
			Magazines with circulation rates 100,001–1,000,000 directed at racial and ethnic minority readers were also included.<br><br>21 magazines identified and 170 articles retrieved.	Portrayals of women with PCOS largely focus on white people.<br><br>Absence of discourse on race and ethnicity, as well as PCOS content directed toward Latinas and African American women.<br><br>Underrepresentation of adolescents with PCOS in magazine articles.
Sang *et al.*, 2022 [75]<br><br>(China)	Social media	Evaluate the effect of WeChat for lifestyle modification on PCOS patients undergoing assisted reproductive technology treatment.	Observational study.<br><br>WeChat group (n= 39)<br>Paper group (n= 40)	Use of WeChat and the suggested lifestyle modifications helped PCOS patients who were undergoing assisted reproductive technology treatment in improving their self-management skills, especially their weight controlling behaviours, and enhancing oocyte quality.
Shreeve *et al.*, 2013 [80]<br><br>(UK)	Wearables	Investigate if there are differences between women with and without PCOS in urinary melatonin levels and the sleep-wake pattern.<br><br>Determine the associations between	Case-control.<br><br>Sleep-wake monitoring (Actiwatch device, wrist-mounted, Phillips Healthcare, Respironics, The Netherlands)<br><br>PCOS group: n = 26<br>Age: 29.8 ± 3.7 years	Serial urine collections over a 24-hour period revealed that night-time melatonin and 8-OhdG levels are significantly elevated in women with PCOS.<br><br>Sleep efficiency (an overall measure of sleep quality) was found to be significantly lower in the PCOS group.

Study ID (Country)	Domain(s)	Aims & Objectives	Study characteristics	Key findings
		circulating melatonin and 8-OHdG; an oxidative stress marker) levels, and sleep quality in women with PCOS.	BMI: 29.3 ± 8.2 kg/m^2<br><br>Control group: n = 26<br>Age: 26.3 ± 5.6 years<br>BMI: 24.6 ± 3.3 kg/m^2	
Stujenske *et al.*, 2023 [53]<br><br>(USA)	Mobile apps	Examine current menstrual cycle tracking technology landscape and explore how these technologies are being used within the context of hormonal and reproductive disorders, including PCOS,	Cross-sectional survey.<br><br>368 respondents:<br>Age: 31.2 ± 5.56 years.<br><br>PCOS diagnosis:<br>- Yes, n= 55 (14.9%)<br>- No, n= 205 (82.9%)<br>- Unsure, n= 7 (1.9%)	Of those with PCOS, 63.6% (n= 35) reported that the use of tracking technologies helped lead to their reproductive disorder diagnoses.<br><br>356 respondents (96.7%) reported using a fertility awareness-based method to track and interpret their menstrual cycle data.<br><br>Most frequently used technologies were urine hormone test/monitor (n= 299, 81.3%), mobile app (n= 253, 68.8%), and temperature tracking devices (n= 116, 31.5%).<br><br>34 different menstrual cycle tracking apps identified. The top five were: Read Your Body, Kindara, Flo, Premom, and Clue.<br><br>Majority of participants reported being either "extremely" (53.5%) or "somewhat" (33.7%) satisfied with the technologies they use.

Study ID (Country)	Domain(s)	Aims & Objectives	Study characteristics	Key findings
				Participants stated that menstrual tracking technologies contributed “a great deal” (38.6%), or “a lot” (35.3%) to their reproductive knowledge.
Suriya Praba *et al.*, 2022 [88]<br><br>(India)	Machine learning	Develop and test a PCOS diagnostic model using machine learning algorithms.	Analysis of the chemical bonding of biological molecules from Raman spectra generated from follicular fluid and plasma samples (n= 100) collected from women going for assisted reproductive technology treatment in Shengjing Hospital, China.<br><br>Age: 23-35 years.<br>PCOS, n= 50.<br>Non-PCOS, n= 50.	AdaBoost classifier model outperformed the other models also tested:<br>- Random Forest,<br>- Multilayer Perceptron,<br>- Decision Tree.<br><br>Raman spectroscopy with advanced machine learning algorithms model can predict PCOS diagnosis with 100% accuracy, using follicular fluid samples.
Tao *et al.*, 2011 [85]<br><br>(China)	Wearables	Determine the features of postprandial glycemic excursions in people with PCOS (using continuous glucose monitoring).	Cross-sectional observational study using continuous glucose monitor (Medtronic MiniMed, CGMS Gold, Northridge, CA, USA)<br><br>PCOS group: n= 45	Those with PCOS had higher ttimes to peak glucose after three meals, and higher amplitude of postprandial glycemic excursions after breakfast.<br><br>No significant differences between the amplitudes of postprandial glycemic excursions after lunch and dinner.

Study ID (Country)	Domain(s)	Aims & Objectives	Study characteristics	Key findings
			Age: 23 ± 3 years<br>BMI: 22.3 ± 4.4 kg/m^2<br><br>Control group: n= 45<br>Age: 24 ± 2 years<br>BMI: 21.4 ± 1.6 kg/m^2	No statistical difference in the mean level of 24-hour blood glucose, standard deviation of blood glucose, and d mean amplitude of glycemic excursion.
Tao *et al.*, 2009 [86]<br><br>(China)	Wearables	Evaluate the characteristics of daily glucose change, insulin sensitivity, and insulin release in people with PCOS with normal glucose tolerance (using continuous glucose monitoring).	Cross-sectional observational study using continuous glucose monitor (Medtronic MiniMed, CGMS Gold, Northridge, CA, USA)<br><br>PCOS group: n= 20<br>Age: 21.8 ± 3.1 years<br>BMI: 21.1 ± 2.6 kg/m^2<br><br>Control group: n= 20<br>Age: 23.0 ± 1.2 years<br>BMI: 20.4 ± 1.5 kg/m^2	Those with PCOS and normal glucose tolerance show:<br>- Shifted peak of glucose-stimulated insulin secretion,<br>- Delayed peak of post-breakfast plasma glucose level,<br>- Decreased peripheral insulin sensitivity with compensated increase of insulin secretion.
Vagenes & Pranic, 2023 [91]<br><br>(Croatia)	Websites	Determine the quality and clarity of information on PCOS available on the internet using validated	Cross-sectional study.<br><br>Search for webpages describing PCOS information in English (not limited to any	The information on the 150 webpages included in the study was accurate; quality and readability not high.

Study ID (Country)	Domain(s)	Aims & Objectives	Study characteristics	Key findings
		tools (DISCERN and EQIP).<br><br>Assess the reading grade level, using standardized tests.<br><br>Assess the accuracy of symptoms described on internet pages about PCOS, by comparison with recent systematic reviews.	specific geographical region), using the top five keywords directly related to PCOS (from Google Trends on February 28, 2022), conducted on Google, Bing, and Yahoo.	Most webpages had a commercial background (58%, n= 87) and originated from the USA (59%, n= 89).<br><br>Other countries of origin: UK (13%, n= 20), India (11%, n= 16).<br><br>Other webpages from: non-profit organizations (29%, n= 44), scientific resources (9%, n= 13), private foundations (4%, n= 6).<br><br>No significant difference in clarity between the three search engines, but possible advantage in using Google as a search engine. The DISCERN median score was higher for Google (compared to Bing and Yahoo), showing a significant difference in quality.
Vasavi *et al.*, 2023 [62]<br><br>(India)	Mobile apps; Machine learning; Wearables	Build a smartphone app to monitor menstrual health, capable of predicting a PCOS diagnosis, using machine learning techniques.	Literature review and mobile app development.<br><br>Literature review identified three PCOS-related apps, which the authors used to compare with their proposed app: Clue, Flo, & AskPCOS.	PCOSMS app developed using Android studio SDK. UI developed using XML. UX developed using Java programming.<br><br>Features included in PCOSMS: Daily exercise, Diet control, PMS calendar, Water intake, Mental hygiene (stress levels monitored through the GSR sensor)

Study ID (Country)	Domain(s)	Aims & Objectives	Study characteristics	Key findings
			Used machine learning for the development process, with a dataset acquired from Kaggle.<br>Includes the use of a wearable device (GSR) to collect stress level information and transmit to the app via Bluetooth.	The Logistic Regression model had the highest precision for PCOS diagnosis among all the other models.
Walter *et al.*, 2022 [77]<br><br>(USA)	Wearables	Study the feasibility of novel advanced wearable sensors to assess the prevalence of sleep disordered breathing in those undergoing IVF.<br><br>Quantify any differences in clinical pregnancy and livebirth rates after treatment among those exposed to sleep disordered breathing.	Prospective observational pilot study.<br><br>Sleep monitoring using a wireless two sensor system (ANNE One, Sibel Health) for general physiological monitoring and diagnosis of sleep disordered breathing.<br><br>Participants (n= 30) undergoing autologous IVF at an academic infertility centre.<br><br>Sleep disordered breathing (apnoea hypopnea index $\geq$5) group, n= 17:	Women with sleep disordered breathing were more likely to carry a primary infertility diagnosis of PCOS (41% vs 8%).<br><br>Sleep disordered breathing was more common among patients with PCOS (87.5% vs 45.5%), but PCOS was not associated with reduced pregnancy rates.

Study ID (Country)	Domain(s)	Aims & Objectives	Study characteristics	Key findings
			Age: 31.4 ± 3.4 years<br>BMI: 28.0 ± 5.3 kg/m^2<br>PCOS: 7 (41%)<br><br>Normal breathing (apnoea hypopnea index <5) group, n= 13:<br>Age: 35.4 ± 5.1 years<br>BMI: 31.6 ± 8.7 kg/m^2<br>PCOS: 1 (8%)	
Wang *et al.*, 2022 [57]<br><br>(China)	Mobile apps	Evaluate the long-term effects of TTM (also known as the Stages of Change Theory) based mobile technology for lifestyle modification on the maintenance of self-managed health-related behaviour changes among women with	RCT.<br><br>Intervention group (n= 51) received same advice as routine care group and had to use the TTM-based smartphone app *Home of PCOS*.<br>Age: 24.72 ± 4.20 years<br>BMI: 25.99 ± 3.87 kg/m^2<br><br>Control group (n= 49) received routine care (i.e. advice on lifestyle):<br>Age: 24.94 ± 4.3 years	Compared to the control group, participants in the intervention group showed a statistically significant decrease for BMI, WC, SAS, and SDS (all *P-values* <.05) at six and 12 months respectively.<br><br>Behaviour stage change of exercise and diet among participants with PCOS was significant at 6 months (c^2=43.032, P<.05) between intervention and control.<br><br>Findings suggest that the TTM-based mobile health app can be applied for lifestyle modification in women with PCOS.

Study ID (Country)	Domain(s)	Aims & Objectives	Study characteristics	Key findings
			BMI: 25.25 ± 3.95 kg/m^2	
Wright *et al.*, 2020 [92]<br><br>(USA)	Websites	Explore the perceived biopsychosocial and medical experiences as presented in stories written by women with PCOS.	Qualitative.<br><br>95 randomly selected stories (from a total of 379 stories), written by women with PCOS on the website www.SoulCysters.com	Three overall themes identified:<br>- Biopsychological struggles and management,<br>- Sociocultural navigation,<br>- Healthcare encounters.<br><br>Significant biopsychological and sociocultural impacts of PCOS on people's daily lives and their medical experiences, which affects their self-image, coping efficacy, and health outcomes.<br><br>There is a need for cultural awareness, providing education, and innovative solutions to healthcare inequities.
Xie *et al.*, 2018 [59]<br><br>(Australia)	Mobile apps	Design and develop an interactive mobile health tool to address the gap in the health information needs of those with PCOS.<br><br>Aims to empower women with PCOS to self-manage their conditions	Five-phase app development process of AskPCOS, which involved: stakeholder consultation, system architecture design and planning, extraction of the evidence-based content from PCOS Guidelines, system prototyping, evaluation and refinement of usability and content quality.	AskPCOS is the first evidence-based, consumer-driven mobile app developed by women and for women with PCOS, utilising innovative technology to empower them and optimise health outcomes.<br><br>Its multilingual content and a not-for-profit cost model facilitate the global adoption of AskPCOS and help to address PCOS information inequities in developing countries.

Study ID (Country)	Domain(s)	Aims & Objectives	Study characteristics	Key findings
		and to promote consistent care.	AskPCOS implements a sociotechnical smart information portal framework for usage-driven design of a dynamic health information gateway.	AskPCOS has extensive data capture capability (through the usage of data gathering functions and backend analytics), which can serve as a powerful research tool enabling and supporting its implementation, monitoring, and evaluation.
Zachurzok-Buczynska *et al.*, 2011 [83]<br><br>(Poland)	Wearables	Assess the risk of cardiovascular disease in adolescent girls with PCOS on the basis of biochemical, echocardiographic, and 24-hour blood pressure monitoring parameters.	Cross-sectional study.<br><br>24-hour blood pressure monitoring (Tracker NIBP2 by DelMar Reynolds Medical, applied to the nondominant arm).<br><br>PCOS group: n= 34<br>Age: 16.0 ± 1.3 years<br>BMI: 24.2 (interquartile range, IQR: 21.3 – 29.0) kg/m^2<br><br>Control group: n= 17<br>Age: 16.2 ± 1.3 years<br>BMI: 22.1 (IQR: 20.9 – 28.8) kg/m^2	Asymptomatic adverse alterations in the cardiovascular system, such as blood pressure and resting heart rate abnormalities, may be present in girls with PCOS even before the age of 18 years.<br><br>Significant differences found between obese and nonobese girls with PCOS.<br><br>Compared with obese controls, obese girls with PCOS had significantly higher:<br>- 24-hour mean blood pressure,<br>- Day mean blood pressure,<br>- Day diastolic blood pressure,<br>- Diastolic blood pressure night-time dip,<br>- Night heart rate.

Study ID (Country)	Domain(s)	Aims & Objectives	Study characteristics	Key findings
Zhang *et al.*, 2023 [74]<br><br>(China)	Social media	Investigate the efficacy of online education through WeChat public account (“Fan says women’s health”) on the lifestyle interventions of women with PCOS.	Letter to the Editor, describing the author’s cross-sectional study.	Those who followed the WeChat account seemed to maintain a healthier lifestyle in both dietary and physical activity assessments.
Zhang *et al.*, 2023 [61]<br><br>(USA)	Mobile apps; Phone-based	Identify abnormal uterine bleeding patterns and their prevalence from the Apple Women’s Health Study (via HealthKit).<br><br>Confirm existing and expected associations between abnormal uterine bleeding patterns, demographics, and medical conditions after confirming the accuracy of tracked data.	Bleeding can be tracked manually via Cycle Tracking in the app or in any third-party menstrual tracking application.<br><br>Sample size: 18,875 women living in the USA (11.7% with PCOS), using an iPhone, and having an iCloud account.<br>Age: 33.0 ± 8.2 years<br>BMI: 29.3 ± 8.0 kg/m^2	Abnormal uterine bleeding was found in 16.4% of participants (n= 3103; 95% CI: 15.9 to 17.0), and:<br>- 2.9% had irregular menses (95% CI: 2.7 to 3.1),<br>- 8.4% had infrequent menses (95% CI: 8.0 to 8.8),<br>- 2.3% had prolonged menses (95% CI: 2.1 to 2.5),<br>- 6.1% had spotting (95% CI: 5.7 to 6.4).<br><br>Black participants (5.9%) had 33% higher prevalence of infrequent menses (PR: 1.33; 95% CI: 1.09 to 1.61). Those with class 3 obesity had 18% higher prevalence of abnormal uterine bleeding compared with those of healthy weight (PR: 1.18; 95% CI: 1.02 to 1.38). Those with PCOS had 19% higher prevalence of abnormal uterine bleeding (PR: 1.19; 95% CI: 1.08 to 1.31) than those without PCOS. Also, an increased prevalence of infrequent menses in those reporting PCOS (PR: 1.44; 95% CI: 1.23 to 1.68).

Study ID (Country)	Domain(s)	Aims & Objectives	Study characteristics	Key findings
Zhu *et al.*, 2012 [81]<br><br>(China)	Wearables	Evaluate the influence of hyperandrogenemia on glucose metabolism in people with PCOS and normal glucose tolerance.	Cohort study.<br><br>Continuous glucose monitor (Medtronic MiniMed, Northridge, CA, USA)<br><br>PCOS with HA: n = 28<br>Age: 22.3 ± 4.3 years<br>BMI: 23.2 ± 5.2 kg/m^2<br><br>PCOS without HA: n= 25<br>Age: 23.4 ± 2.8 years<br>BMI: 23.5 ± 4.1 kg/m^2	In people with PCOS with normal glucose tolerance, those with HA had:<br>- higher mean blood glucose levels,<br>- a greater percentage of time with hypoglycemia during the period of continuous glucose monitoring (compared with non-HA patients of similar age and BMI).<br><br>Serum levels of LH and dehydroepiandrosterone sulfate were significantly higher in the HA group.
Zigarelli *et al.*, 2022 [89]<br><br>(USA)	Machine learning	Develop self-diagnostic prediction models for PCOS using machine learning techniques.	Retrospective observational study.<br><br>Dataset (publicly available) collected from 10 different hospitals in Kerala, India (n= 526).<br>Age: 31 (IQR 27 – 35) years<br><br>PCOS: n= 170<br>Age: 29 (IQR 26.25 – 33) years	In the model for potential patients (users who do not have medical test results available) – prediction accuracy ranged from 81% - 81.5% with subgroups and 82.5% without subgroups.<br><br>In the model for clinical providers (or users who have access to medical records and test results – prediction accuracy ranged from 87.5% - 89.8% with subgroups and 90.1% without subgroups.

Study ID (Country)	Domain(s)	Aims & Objectives	Study characteristics	Key findings
			BMI: 25.1 (IQR 26.25 – 33) Kg/m^2<br><br>Non-PCOS: n= 356<br>Age: 32 (IQR 28 – 36) years<br>BMI: 23.61 (IQR 21.37 – 26.13) Kg/m^2<br><br>The authors adopted:<br>- the CatBoost method for classification,<br>- K-fold cross-validation for estimating the performance of models,<br>- SHAP values to explain the importance of each variable.<br><br>Subgroup study: k-means clustering and CPA used to split the dataset into two distinct BMI subgroups to compare:<br>- the prediction results,<br>- the feature importance between the two subgroups.	

Study ID (Country)	Domain(s)	Aims & Objectives	Study characteristics	Key findings
			Subgroup 1:<br>PCOS: n= 76<br>Age: 31 (IQR 28 – 35) years<br>BMI: 22.15 (IQR 20.29 – 23.9) Kg/m^2<br><br>Subgroup 2:<br>PCOS: n= 94<br>Age: 31 (IQR 27 – 35) years<br>BMI: 26.75 (IQR 25.1 – 28.98) Kg/m^2	

Key: 8-OHdG: 8-hydroxy-2-deoxyguanosine; API: Application programming interfaces; BMI: body mass index; CI: confidence intervals; CPA: principal component analysis; DISCERN: 16-item DISCERN questionnaire, an instrument for judging the quality of written consumer health information on treatment choices; EQUIP: 36-item EQIP (ensuring quality information for patients) tool; FSH: follicle-stimulating hormone; GSR: galvanic skin response; GPs: general practitioners; HA: hyperandrogenism; HCP: healthcare professionals; IQR: interquartile range; IVF: *in vitro* fertilization; LH: luteinizing hormone; MVPA: moderate to vigorous physical activity; OGTT: oral glucose tolerance test; OR: odds ratio; PCOS: polycystic ovary syndrome; PCOSMS: PCOS Monitoring System; PMS: premenstrual syndrome; PR: prevalence ratio; QUADAS-2: Quality Assessment of Diagnostic Accuracy Studies; RCT: randomized controlled trial; SAS: Zung's Self-Rating Anxiety Scale; SDK: software development kit; SDS: Zung's Self-Rating Depression Scale; SHAP: Shapley Additive Explanations; TCM: Traditional Chinese Medicine; TTM: transtheoretical model; UI: user interface; UX: user experience; WC: waist circumference; XML: Extensible Markup Language.
