# Supplementary material for: Polycystic Ovary Syndrome and the Internet of Things: A Scoping Review
Source: Healthcare (Basel). 2024 Aug 21;12(16):1671. doi: 10.3390/healthcare12161671 (PMC11354210; doi:10.3390/healthcare12161671)
Supplement: Supplementary file 1 [file healthcare-12-01671-s001.zip › Supplementary_File_S4_Participants_voice.pdf]

Study ID (Country)	Study characteristics	Aims & Objectives	Direct Quotes
Cowan *et al.*, 2023 [97]<br><br>(Australia)	Online cross-sectional survey.<br><br>Participants: n= 1167<br>Age (mean ± standard deviation): 32 ± 7 years<br>BMI: 34.3 ± 8.9 kg/m^2	Identify types and sources of dietary and physical activity interventions used by women with polycystic ovary syndrome (PCOS).<br><br>Characterize the behavioural and cognitive self-management strategies that they use when they implement dietary or physical activity interventions.	*"More than half of participants in this study used the internet and social media as their primary source of diet and physical activity information."*<br><br>*"Findings suggest that online information may promote inaccurate and ineffective lifestyle advice and emphasize the need to increase engagement with qualified health professionals."*
Ismaylova & Yaya, 2022 [98]<br><br>(Canada)	Semi-structured interviews.<br><br>Participants: n= 25<br>Age range: 18 – 63 years	Explore the lived experiences of women with PCOS in Canada and identify barriers and facilitators for their symptom management.	*"In the group that I'm reading now, people have the same issues which I know and it's so nice, the group is actually what I find support in now. I really enjoy reading it and being able to connect with the people. Before that I did not have any support. I'm actually feeling much better now. I was actually really depressed about it. I didn't have a way out to speak to anyone. No one would actually understand it. So now with that group, it's definitely much better."*
Copp *et al.*, 2021 [17]<br><br>(Australia)	Semi-structured interviews.<br><br>Participants: n= 26	Explore the experience of managing PCOS for both clinicians and women with PCOS.	*"I was in the PCOS Australia group and I just found connecting to other women really helpful."*<br><br>*"I'm on a support page on Facebook, which has its ups and downs really, because you don't really get a lot of information…So*

Study ID (Country)	Study characteristics	Aims & Objectives	Direct Quotes
	Age range: 18 – 45 years (mean age: 29.3 years)<br><br>Clinicians: n= 36		*something that might affect one person might not affect you and so you really have to try and not base yourself on other people."*<br><br>*"Just the potential stigmatization, people reading information online that actually doesn't really apply to them. Because it's such a wastebasket."*
Lim *et al.*, 2021 [44]<br><br>(Australia)<br><br>plus Ee *et al.*, 2020 [45]	Focus groups & semi-structured individual telephone interviews.<br><br>Participants: n= 10<br>Age: 36.1 ± 7.24 years<br><br>Body mass index (BMI): 36.38 ± 7.8 kg/m^2	Explore the health literacy and the needs of those living with PCOS regarding lifestyle and weight management, in order to inform research and clinical practice.<br><br>Explore the experiences of those living with PCOS, in order to highlight gaps in care and inform the provision of patient-centred healthcare.	*"I think reading some of those posts and going through the comments and stuff it does give you a sense of relatability."*<br><br>*"I do sometimes skip over them because maybe I don't want to hear too much negative about that"*<br><br>*"I don't know anyone else that has PCOS. Sometimes I'll talk to my girlfriends and stuff and they just don't get it."*<br><br>*"If I go on and someone's posted something and I go I can relate to that, if I click in and see comments I just start to get anxious"*
Holton *et al.*, 2018 [99]<br><br>(Australia)	Online discussion group (private group on the social media platform Facebook).<br><br>Participants: n= 13	Identify the fertility and childbearing concerns of women with PCOS, as well as their related information needs and preferences.	*"I have found information but it was on the Internet. ... [and from] other women with PCOS (family and friends) [and] I just joined a PCOS support group on Facebook."*<br><br>*"I find government/doctor type fact sheets lacking in information/specificity but find academic papers too information*

Study ID (Country)	Study characteristics	Aims & Objectives	Direct Quotes
	Age range: 22 – 43 years (mean age: 30.8 years)		*dense/specific. I want something in between which is just a good overview of the known science, treatment options, symptoms, etc all in one place."*
Williams *et al.*, 2016 [100]<br><br>(UK)	Photovoice investigation.<br><br>Participants: n= 9<br>Age: 20 – 45 years *(5 did not disclose age).*	Explore the impact of PCOS in women's day-to-day life.	*"I have learnt by attending PCOS conferences that eating is very important, especially eating the right things."*<br><br>*"I've learned so much on tumblr—way more than through doctors. I'm afraid to take Metformin (though I know it's coming) because it can make you feel so sick, and it was only through tumblr that I was able to hear about alternatives. Talking to other women with it is INVALUABLE."*
Holbrey & Coulson, 2013 [101]<br><br>(UK)	Online questionnaire with both closed and open-ended items.<br><br>Participants: n= 50<br>Age range: 28 – 38 years (mean age: 33.6 ± 5.36 years)	Explore the views and experiences of women living with PCOS who participate in an online support group discussion for issues related to PCOS:<br>- Does it empower them?<br>- If so, how?<br>- Do any potentially disempowering processes arise from their online interaction?	*"It is fantastic to be able to discuss issues and concerns with people who completely understand what I am talking about, other than the support groups there is a lack of support as people cannot fully understand the condition unless they too suffer with it themselves"*<br><br>*"Makes you feel more anxious when you read about other people's concerns and more severe problems – either you berate yourself for worrying about nothing or feel that the worst is yet to come."*

Study ID (Country)	Study characteristics	Aims & Objectives	Direct Quotes
Avery & Braunack-Mayer, 2007 [102]<br><br>(Australia)	In-depth qualitative interviews.<br><br>Participants: n= 10<br>Age range: 28 – 38 years (mean age: 32.4 years)	Explore the information that women with PCOS want to know about their condition and the impact such information may have for future treatment and health outcomes.	*"Finally when the Internet was connected in late 1997, that was the explosion when I connected up to the Internet and thought "My God! – look what we've discovered!"... I've got that many bookmarks on the Internet. Tried to just become informed... Online is good, but I understand that so many people it's not for them."*<br><br>*"I like the way I can access the information, that I can do it all of the time, night and day... Because it's accessible, because there's lots of stuff on there, look up more than one thing. Because it's easy for me to use... I can do it privately as well. At least this way I can explore by myself."*
